# Insulin-like growth factor-1 deficiency and metabolic syndrome

**DOI:** 10.1186/s12967-015-0762-z

**Published:** 2016-01-06

**Authors:** G. A. Aguirre, J. Rodríguez De Ita, R. G. de la Garza, I. Castilla-Cortazar

**Affiliations:** Escuela de Medicina, Tecnologico de Monterrey, Avenida Morones Prieto No. 3000 Pte. Col. Los Doctores, 64710 Monterrey, Nuevo León Mexico; Fundación de Investigación HM Hospitales, Madrid, Spain

**Keywords:** Lipid metabolism, Insulin resistance, Dyslipidemia, Aging, Oxidative stress, GH/IGF-I axis, Obesity, Mitochondrial dysfunction, Cellular protection

## Abstract

Consistent evidence associates IGF-1 deficiency and metabolic syndrome. In this review, we will focus on the metabolic effects of IGF-1, the concept of metabolic syndrome and its clinical manifestations (impaired lipid profile, insulin resistance, increased glucose levels, obesity, and cardiovascular disease), discussing whether IGF-1 replacement therapy could be a beneficial strategy for these patients. The search plan was made in Medline for Pubmed with the following mesh terms: IGF-1 and “metabolism, carbohydrate, lipids, proteins, amino acids, metabolic syndrome, cardiovascular disease, diabetes” between the years 1963–2015. The search includes animal and human protocols. In this review we discuss the relevant actions of IGF-1 on metabolism and the implication of IGF-1 deficiency in the establishment of metabolic syndrome. Multiple studies (in vitro and in vivo) demonstrate the association between IGF-1 deficit and deregulated lipid metabolism, cardiovascular disease, diabetes, and an altered metabolic profile of diabetic patients. Based on the available data we propose IGF-1 as a key hormone in the pathophysiology of metabolic syndrome; due to its implications in the metabolism of carbohydrates and lipids. Previous data demonstrates how IGF-1 can be an effective option in the treatment of this worldwide increasing condition. It has to distinguished that the replacement therapy should be only undertaken to restore the physiological levels, never to exceed physiological ranges.

## Background

Consistent data from multiple studies (in vitro and in vivo) demonstrate the association between IGF-1 deficit and deregulated lipid metabolism, cardiovascular disease (CVD), diabetes, and altered metabolic profile of diabetic patients. On the other hand, metabolic syndrome (MetS) is a constellation of symptoms that implies a higher risk for CVD and type 2 diabetes (T2D), increasing the morbidity and mortality of these patients. As it is a syndrome clustering different features, the common causative aetiology is yet unknown. Nevertheless, the insulin resistance and abdominal adiposity seems to be essential in the pathophysiological process, and for this reason, based on the information available, we propose IGF-1 as a key hormone in the pathophysiology of metabolic syndrome due to its implications in the metabolism of carbohydrates and lipids. Accumulated evidence shows that IGF-1 can be an effective option in the treatment of this prevalence-increasing condition, as we shall further explain in detail.

Therefore, in this review our aim is to bring all IGF-1 information regarding metabolism together with the objective of offering clear insight into the issue—this way clarifying how miss-regulation of the GH/IGF-1/insulin axis can lead to metabolic disorders such as MetS and diabetes; introducing how obesity and insulin resistance (initiating factors for the onset of MetS and diabetes) may be opposed by recombinant-human-IGF-1 (rhIGF-1) treatment. Firstly, the concept of metabolic syndrome and its different definitions will be reviewed, together with the symptoms or risk factors associated. Secondly, IGF-1’s molecular structure and known actions with emphasis on metabolic actions—which will be discussed in detail. To continue, IGF-1 and IGF-1 binding proteins will be linked to parameters of metabolic syndrome, diabetes, insulin resistance, and obesity. Lastly, IGF-1 will be proposed as valid optional treatment for patients with MetS in which diet and exercise failed due to genetic traits.

## Metabolic syndrome

For many decades, the concept of “clustering” metabolic disorder and CVD risk factors has been widely discussed. Table 1 summarises historical definitions and evolution of MetS diagnosis. However, the term “Metabolic Syndrome” has become commonly used since its inception by the “Executive Summary of the Third Report of the National Cholesterol Education Program” (NCEP) in 2001 [1]. Since then, many different concepts and definitions have been proposed. Hence, it was not until 2009 when a harmonised definition was finally described [2]. According to this definition, a diagnosis of MetS is made when 3 of the following 5 risk factors are present: enlarged waist circumference with population and country-specific criteria; elevated triglycerides (defined as ≥150 mg/dL), decreased High Density Lipoprotein (HDL) (ranges below 40 mg/dL in men and 50 mg/dL in women), elevated blood pressure (defined as systolic blood pressure above 130 mmHg or diastolic blood pressure above 85 mmHg) and elevated fasting glucose (defined as blood glucose above 100 mg/dL). This definition includes those patients that are taking medication to manage hypertriglyceridemia, low HDL, hypertension and hyperglycemia [2].

**Table 1:** History definitions for metabolic syndrome

Definitions	Insulin	Body weight	Blood pressure	Lipids	Glucose	Others
Kylin (1923)			Hypertension		Hyperglycaemia	Gout
Vague (1947)	Diabetes	Central obesity		Atherosclerosis		Gout
EASD (1965)	Hyperglycaemia	Obesity	Hypertension			
Raeven (1988) “syndrome X”	Insulin resistance		Hypertension	High TG levels, low HDL-c	Glucose intolerance	
Kaplan (1989) “The Deadly Quartet”		Upper body obesity	Hypertension	Hypertriglyceridaemia	Glucose intolerance	
WHO (1998)	High insulin levels, IFG or IGT Plus any 2 of the following	Abdominal obesity: WHR >0.9 for men and >0.85 for women, BMI >30 kg/m^2^	BP ≥160/90 mmHg	TG ≥150 mg/dL, and/or HDL-c <35 mg/dL in men, <39 in women		Microalbuminuria: urinary excretion rate >20 mg/min or albumin: creatinine ratio >30 mg/g
EGIR (1999)	Plasma insulin > 75th percentile Plus any 2 of the following	WC ≥94 cm for men and ≥80 for women	BP ≥140/90 mmHg or antihypertensive medication	TG ≥150 mg/dL and/or HDL-c <39 mg/dL in men or women	IGT or fasting glucose 6.1 mmol/L, but no diabetes	
NCEP-ATPIII (2001)	None 3 or more of the following	WC ≥ 102 cm for men and ≥ 88 cm for women	BP ≥130/85 mmHg	TG ≥150 mg/dL or HDL-C <40 mg/dL for men, <50 mg/dL form women	FPG >110 mg/dL (includes diabetes)	
AACE (2003)	IGT or IFG Plus 2 of the following	BMI ≥25 kg/m^2^	BP ≥130/85 mmHg	TG ≥150 mg/dL and HDL-c <40 mg/dL for men, <50 for women	IGT or IFG (but no diabetes)	Other features of insulin resistance^a^
IDF (2006)	None	Central obesity as defined by ethnic/racial Plus 2 of the following	BP ≥130 mmHg systolic or ≥85 mmHg diastolic, or under treatment	TG ≥150 mg/dL or under treatment, or HDL-c <40 mg/dL for men, <50 mg/dL for women	IFG ≥ 100 mg/dL (includes diabetes)	
Harmonization definition (2009)	None 3 or more of the following	Enlarged WC according to population and country specific criteria	BP ≥130 mmHg systolic or ≥85 mmHg diastolic, or under treatment	TG ≥150 mg/dL and HDL-c <40 mg/dL for men, <50 for women, or under treatment	IFG ≥ 100 mg/dL, or under treatment	

*IFG* impaired fasting glucose, *IGT* impaired glucose tolerance, *WHR* waist-to-hip ratio, *BMI* body mass index, *WC* waist circumference, *BP* blood pressure, *HDL-c* high density lipoprotein cholesterol, *TG* triglycerides

^a^Family history of type 2 diabetes, polycystic ovary syndrome, sedentary lifestyle, advancing age, and ethnic groups susceptible to type 2 diabetes

In general MetS continues to be a clustering of symptoms that seem to play a major role as CVD and T2D risk factors, raising the necessity of encouraging these patients to pursue lifestyle changes. Moreover, the harmonised definition criteria was found to be a better predictor of CVD than each of its separate components or the Framinghan Score—this was not applicable for T2D [3].

The prevalence of MetS is difficult to establish since it depends on the definition [1, 2, 4–6] and the composition of the population being studied [7–29]. To dissolve this issue different studies have been undertaken in order to estimate the prevalence of MetS. Nowadays we can understand that sex, age, race and ethnicity, in the context of socioeconomic status and lifestyle (tobacco, alcohol, education, physical exercise, unbalanced diet, etc.) impacts directly in its prevalence [7, 8, 10, 27– 36]. Furthermore, it is well known that it is increasing worldwide [37], this is thought to be related to the westernisation of lifestyle habits [38, 39].

It is well known that MetS represents an assortment of factors that increase the risk of CVD [11] and T2D by different means [11, 40–55]. Obesity and insulin resistance are associated with endothelial dysfunction, sympathetic nervous system hyperactivity, and hyperleptinaemia, all of which can lead to hypertension. Furthermore, insulin resistance can lead to abnormal lipid profiles, like low-HDL and high triglyceride (TG) levels. These two factors can increase the risk for CVD [56–60], nonetheless there is some controversy about the role of TG levels in the CVD development [57]. In summary, the majority of the studies have found that patients with MetS are at increased risk for developing CVD [11, 37, 39–55]. Additionally, as previously mentioned, a recent study has described that MetS -taking the harmonised definition criteria for diagnosis—is a better predictor for CVD than the sum of each of its separate components or the Framingham Score. Other studies have found that the more components of the MetS are present, the greater the risk of developing CVD [42, 53, 61].

Moreover, MetS is also a good predictor for the development of T2D [62–64]. Insulin resistance, hyperinsulinemia, dyslipidaemia, and obesity precede the progression to T2D in 75–85 % of patients [65], and the presence of MetS increases up to fivefold the risk for T2D compared to individuals without MetS [66, 67]. This risk is increased up to 6-7-fold, if insulin resistance is present [54].

Other conditions that have been associated with MetS are strongly related with insulin resistance and adiposity, namely, non-alcoholic fatty liver disease (NAFLD), polycystic ovarian syndrome, obstructive sleep apnoea (OSA), hypogonadism, lipodystrophy, and microvascular disease. Moreover, the presence of NAFLD is a robust predictor of MetS [68]. Liver fat also correlates to each of the components of MetS [69]. In the case of OSA, there is evidence—adjusting for obesity—that individuals with this alteration are more likely to develop MetS than those without OSA [70–72]. Also, sleep disorders have been associated with weight gain and insulin resistance [73–77]. In the case of microvascular disease, some studies relate it to MetS, independent to the presence of T2D [78–86], but further studies are needed to ensure these results.

## Insulin-like growth factor-1

Insulin-like growth factor 1 (IGF-1) is a 70-aminoacid polypeptide hormone with endocrine, paracrine, and autocrine effects, which shares structural homology (>60 %) with IGF-2 and proinsulin [87, 88]. It is mainly produced by the liver (accounting for ≈75 % of circulating IGF-1) secondary to growth hormone (GH) and insulin endocrine stimulation in the liver. Conversely, IGF-1 acts to provide an inhibitory feedback signal on GH secretion in the hypothalamus by stimulating somatostatin production in the pituitary [89–91]. IGF-1 is also produced locally in all bodily tissues [92]. IGF-1 availability is tightly regulated by the so-called insulin-like growth factor binding proteins (IGFBPs), which may act by increasing IGF-1 half-life, from minutes to hours (most commonly by forming a tertiary complex with Acid-Labile Subunit and IGFBP3), however blocking its binding to the insulin-like growth factor 1 receptor (IGF-1R) [93–95]. IGFBPs can also act to guide IGF-1 to specific tissues, or even to inhibit or potentiate IGF-1 actions by acting as an independent substrate for the IGF-1R and/or other specific membrane, intracellular or nuclear receptors [93–95]. To date there have been described 6 high affinity IGFBPs [93–95]. Moreover, insulin-like growth factor binding protein related proteins (IGFBPrPs) have been recently characterised, which aid the metabolic effects of the hormone but their role remains unclear [96, 97]. Relevant roles of each specific IGFBPs will be further discussed when applicable to metabolism since there is a huge emerging world of IGFBPs independent actions.

IGF-1 can act over its putative receptor (IGF-1R) or it can also bind to the insulin receptor (IR), albeit with less affinity [98–100]. In addition, there is a hybrid receptor with components of the IR (one α and one β-chain) and the IGF-1R (one α and one β-chain), to which both insulin and IGF-1 can bind to, but with less affinity than that of their putative receptors [98–100]. All of these receptors have tyrosine kinase activity, hence are natural and potent activators of the Akt pathway [98–100].

IGF-2 actions have been poorly characterised, however relevant roles have been determined for foetus development and cerebral protection [101, 102]. IGF-2 can act over its own receptor (IGF-2 receptor—IGF-2R), which is a manose-6-phosphate transmembrane protein with undetermined actions—it is thought that it acts as a scavenger receptor by sequestering IGF-2 and IGF-1 from the extracellular medium and targeting them for destruction [103]. Nonetheless, it seems that it could have intracellular targets instead of only being proteolysed. As of recent discovery IGF-2R shows to activate G_αq_ proteins within cardiomyocites [104]. Additionally, IGF-2 can also act over IGF-1R, IR and hybrid receptors but with reduced affinity [103].

In the last decades, IGF-1 has been implicated in many physiological actions, among others: tissue growth and development, proliferative, lipid metabolism, pro-survival/anti-aging, anti-inflammatory, anabolic, and antioxidant with neuro- and hepatoprotective properties [105–116]. IGF-1 exerts protective effects over mitochondria by preserving it from the oxidative damage generated by augmented metabolism, and increasing ATP synthesis and reducing intramitochondrial production of free radicals [105–107].

### Insulin receptor, insulin-like growth factor-1 receptor and insulin resistance signalling

As a brief review of insulin signalling and its resistance molecular basis: insulin and IGF-1 receptors (IR and IGF-1R) are tyrosine kinases. As such they attract molecules containing a Src-homolgy 2 (SH2) domain (several docking sites for phosphorylated tyrosines). The most often and potent ones attracted are the insulin receptor substrates 1/2 (IRS1/2)—although there are 6 found to date- (not to forget that Shc proteins, p60dok, Cbl, APS, and Gab-1 are also recruited to activated IRs). These provide additional tyrosine residues to be phosphorylated by the tyrosine kinase domain of the activated receptor that will attract further molecules containing SH2 domains or plekstrin homoly (PH) domains, these last will anchor IRS to phosphoinositides on the cell membrane. When PI3K and its regulatory proteins, p85 and p110, are recruited by IRS, they will further recruit and activate PDK1 (PIP_3_-dependent kinase 1), Akt (PKB), mTORC2, S6 kinases and PKC; all leading to augmented glucose transport, glycogen and protein synthesis. Zick and colleagues [117] have elegantly summarised recent evidence that show how IRS also possess serine residues that can be phosphorylated. When this happens tyrosine phosphorylation becomes less likely to happen. This is, in a certain way, a termination pathway to uncouple the insulin signalling. There are other mechanisms to terminate the insulin signalling that include lipid and protein phosphatases along the cascade and controlling mechanisms; long-term regulation includes transcription inhibition of the IR and proteolysis by ubiquitination. One convergent pathway activated by IGF-1R and IR is the mTORC1 and mTORC2 signalling. It is widely known they both posses serine and threonine phosphorylation capability. However, it has been recently described that mTORC2 also possesses tyrosine phosphorylation capacity [118], and that it phosphorylates tyrosines on IRS and tyrosine kinases in both receptors, IGF-1 and IR, thus reinstituting the signal of the activated receptors [118]. Whilst mTORC1 activates S6 kinase, which phosphorylates serine residues on IRS which in turn uncouples IRS from the receptor and its substrates, mTORC2 can reactivate this signalling.

Complementary to the above, it has been thought for a long time that only supraphysiological concentrations of IGF-1 are able to activate the IR, as will be further discussed in this manuscript. However, the exact mechanism by which IGF-1 improves insulin signalling has not yet be explained (other than indirect actions through lipid clearance from the bloodstream by inhibiting GH (lipolysis on adipocytes) and FFA uptake by muscles; all these mechanisms are collected below). We now propose a feasible mechanism: Denley and colleagues [119] have beautifully designed a study where they demonstrate how IR has a splice variant lacking exon 11 which confers the receptor affinity for IGF-1 and IGF-2. In this way, IGF-1 gains the ability to stimulate the IR, and without activating the tyrosine kinase domain, recruits IRS-2. Complementarily, IGF-1R preferentially activates IRS-2 [120] as it was found that IRS-2 contains a KLRB domain that functions to block the tyrosine kinase domain in the cytoplasmic region of the IR, and that such does not happen in the IGF-1R. Thus suggesting a specificity for IGF-1R. It has been found, using specific knock out (KO) mice and cultures, different specific activities for IRS-1 and IRS-2, additional further complexity comes with tissue-specific roles. For example, in muscle, IRS-1 is more related to glucose uptake whereas IRS-2 stimulates the MAPK pathway [121]. In the liver, they both have metabolic regulation actions, but IRS-2 has a more profound role in lipid metabolism [121]. Additional complexity, and in accordance with IGF-1 secretion patterns, appeared when researchers found that IRS-1 was found more active in post-prandial states contrary to IRS-2 in fasting states [121]. Even more interesting is the fact that Shc and PLC were found to only interact with IRS-2 [121]. Recall that Shc ultimately activates the MAPK pathway, while PLC has more metabolic effects including GLUT4 translocation. IGF-1 displays more binding sites for SHP2 (a phosphatase related to growth) and seems more prone to recruit Cbl [121] (an E3 ligase that targets the receptor for ubiquitination and destruction) and thus may explain a different regulatory mechanism not mediated by serine phosphorylation, and thus not so sensitive to metabolic derangements. Intriguingly, another interesting research lead to the discovery of a differential role for IRSs in apoptosis, suggesting an antiapoptotic effect for IRS-2 [122] which is consistent with known differential roles of IGF-1 and insulin.

Taking all this data together it seems logical or appropriate to conclude that, because IGF1-R has a different signalling pathway that can maintain lipid oxidation in the liver, FFA uptake in muscle, and activates mTORC1 could reactivate IR through tyrosine kinase activity on IRS, thus displacing serine phosphorylation, reinstituting insulin signalling. Also since most of serine inhibiting phosphorylation occurs in IRS-1, it renders IGF-1R a rescue pathway to reinstitute insulin sensitivity. Because IGF-1 is normally found at low levels in MetS and T2D, maybe because of insulin cessation to inhibit IGFBP-1 production by the liver and because of decreased liver IGF-1 secretion by insulin stimulation, as insulin resistance prevails in the liver. Consistent with the evidence presented we suggest a positive effect towards re-establishing IGF-1 levels by substitutive therapy only to physiological levels, never above them.

## Insulin-like growth factor-1 metabolic effects

At a first glance, IGF-1 has historical fame for being a growth and differentiation factor, however, a number of growth-unrelated actions have been recently unravelled [123]. From our perspective IGF-1, GH, and insulin conform a finely regulated axis that inform cells about the nutritional status of the organism so that they can either undergo apoptosis/senescence/quiescence or, to the contrary, grow and differentiate. Parallel to this signal, potent protective effects have been attributed to this hormone, thus, besides signalling abundance and growth, it provides the protection against the possible deleterious effects of augmented metabolism. Likewise, anti-inflammatory actions of IGF-1 [124] can be regarded as a crucial factor protecting tissues from the deleterious effects of pro-inflammatory mediators in chronic disorders such as obesity. It has been well established that pro-inflammatory cytokines produced by the adipose tissue in obesity affect normal nutrition-related signalling, establishing the progression to MetS and ultimately to T2D [125]. Additionally, it is now known that pro-inflammatory cytokines also hijack IGF-1 intracellular signalling by phosphorylating serine residues on insulin related substrate (IRS) molecules and hence impeding their binding to IGF-1R [124]. This results in a blockade of IGF-1 beneficial actions [124, 126, 127]. Under this scenario, a correlation between IGF-1 and MetS can be established.

When caloric restriction is present, mammals synthesise less IGF-1 and its synthesis in the liver is refractory to GH stimulation [128–130]. This process functions to limit growth and protein synthesis when nutrient availability is compromised. After a meal, GH responsiveness and IGF-1 synthesis is reinstituted [105, 106]. When inadequate carbohydrates are ingested, the decrease in portal vein insulin concentration leads to a reduction in IGF-1 synthesis by the liver [129, 131]. In pathophysiological states, including increased insulin resistance, the hybrid receptor number is changed significantly, thus potentially abrogating the chance for IGF-1 to alter glucose metabolism [129, 132, 133]. It is important to mentions that IGF-1 receptors are expressed ubiquitously [99]. This means that their actions can occur in all cell types, stimulating fat, carbohydrate, and protein metabolic coordination, as we shall explain in detail herein. IGF-1 possesses both, GH-like actions and insulin-like actions, whose effects in vivo depend on the dosage, length of treatment, and even route of administration [134, 135]. However, GH can also exert metabolic actions independent from IGF-1 generation in the liver via activation of the phosphoinositide 3-kinase (PI3 K) and IRS pathways [98]. In this way, GH and insulin act in symphony with IGF-1 to produce a coordinated response. Supported by an increasing number of studies these effects suggest the involvement of IGF-1 in metabolism coordination [136].

### Insulin-like growth factor-1 and carbohydrate metabolism

IGF-1 can promote glucose uptake in certain peripheral tissues [137–140] in the magnitude of 4–7 % from that of insulin [141, 142]. In addition, exogenous IGF-1 administration has been shown to reduce serum glucose levels [95, 143, 144], not only in healthy individuals [140, 145–147], but also in those with insulin resistance [148, 149], type I [150–152], and T2D [153–155]. An interesting experiment shows how, in the presence of insulin resistance, there is up-regulation of the insulin/IGF-1 hybrid receptor expression in both, muscle and fat [133, 156]. It is important to bear in mind that IGF-1 serum concentration is 100-fold greater than insulin, however when bound to IGFBPs its biological activity is modulated and in its free-unbound form presents different effects [99, 157]. High doses of IGF-1 administration typically results in hypoglycaemia despite the potent suppression of circulating insulin concentrations it triggers [134, 135, 158]. Even though this effect may be mediated by the insulin receptor, experimental KO mice for the insulin receptor gene show a potent glucose lowering effect of IGF-1, indicative that the hypoglycaemic effect is also mediated, in part, by its own IGF-1R. However, this study was undertaken in 1–3 day old mice, as without IR they were not viable, and thus, results are not conclusive [159].

Berryman and colleagues revised IGF-1 actions on obesity, and concluded that this molecule possesses direct effects on muscle glucose uptake [160]. Moreover, KO mice for liver IGF-1 gene developed muscle insulin resistance (it is important to mention that skeletal muscle myocytes express high number of IGF-1R), showing an increase in insulin concentrations and a substantial decrease in the insulin-induced autophosphorylation of the insulin receptor and IRS in skeletal muscle (being normal in liver and white adipose tissue). This effect was efficiently reverted by IGF-1 administration [161]. Such results could indicate that hepatic derived IGF-1 plays a crucial role in skeletal muscle insulin signalling and glucose uptake. In murine studies, deletion of the IGF-1R in skeletal muscle resulted in glucose intolerance issues ultimately leading to T2D at an early age, because, although they express insulin receptors, they cannot form hybrid or IGF-1 receptors [162]. When these mice where administered IGF-1 they showed lowered fasting glucose, and because no functional IGF-1R is present in muscle, such effect is believed to be due to renal gluconeogenesis suppression [129, 163]. Our group, working with partially deficient IGF-1 mice, has demonstrated that the liver is capable of expressing IGF-1R (which under physiological conditions it does not [99]) as a “defence” mechanism (peer review). If the abovementioned is occurring, it could mean that IGF-1 administration could also lower glucose levels by suppressing hepatic gluconeogenesis, as well as improving insulin signalling in this organ, leading to IGFBP-1 suppression and overall improvement of the IGF-1/GH/insulin axis. Furthermore, additional performed studies suggested [164] that genetic expression of enzymes involved in glucose and lipid homeostasis together with cholesterol transport are altered (as we shall further explain in detail later on).

IGF-1 reduces serum GH levels (via somatostatin negative feedback in the pituitary) which in turn suppresses GH actions in the liver, thus enhancing insulin actions in this organ [165]. In both, fat and liver, GH stimulates the synthesis of p85 subunit of PI3K [166] leading to the suppression of p110 subunit activity and, thus, antagonising insulin’s actions [167]. Therefore, IGF-1 may indirectly modulate carbohydrate metabolism through GH suppression and enhancement of insulin action.

During postprandial periods there is an increase in free circulating IGF-1 via insulin-induced suppression of IGFBP-1 secretion [168–170], which sequesters free IGF-1 making it unavailable. IGFBP-1 gene is transcriptionally regulated by insulin in the liver [169]. This change in available IGF-1 may be (it is difficult to extrapolate data from altered IGF-1/GH concentrations from animal models, as each author measures IGF-1 levels using different methods) adequate for fatty acid (FA) oxidation in muscle, suppression of GH, stimulation of glucose transport into muscle [171, 172], and lastly for the suppression of renal gluconeogenesis in mice [163].

Besides the already discussed actions of IGF-1 on glucose, it also has an indirect glucose-lowering effect secondary to its ability in increasing FA oxidation in muscle (will be further discussed in detail within the *IGF*-*1 and lipid metabolism* section). Such ability produces a decreased FFA flux in the liver and hence insulin signalling is improved, being now able, such signalling to suppress hepatic glucose output [166].

IGFBPs are also hypothesised to play a role in glucose metabolism. IGFBP-1 regulates glucose levels through its effect on free IGF-1. IGFBP-2 actions are linked to insulin, although only in cases of hyperinsulinemia [173] where it seems to play a role on adipocyte autocrine control [174]. It has been reported that IGFBP-3 binds to a nuclear receptor, 9-cis retinoic acid receptor-alpha (RXR-α), which interacts with peroxisome proliferator activated receptor-gamma (PPAR-γ), a nuclear protein involved in the regulation of glucose and lipid metabolism [175, 176]. A transgenic mice study stated that the overexpression of IGFBP-3 is associated with impaired glucose tolerance [177, 178]. Also, IGFBPrPs have been associated with insulin resistance and fasting glucose levels [179, 180].

In a recent investigation undertaken by our group, adult mice with partial IGF-1 showed a decrease in the expression of genes involved in glucose metabolism (phosphoenolpyruvate carboxylase-1, glucose-6-phosphatase, pyruvate dehydrogenase kinase isoenzyme-4, and ATP-citrate lyase), resulting serious hyperglycaemia [164]. Such genetic alterations were all reverted by low doses if IGF-1 replacement therapy for only 10 days. Interestingly, it is well accepted that insulin increases the expression of glucose-6-phosphatase and phosphoenolpyruvate carboxylase-1. Results in the study demonstrate that IGF-1 induces the opposite effects since the IGF-1 deficit reduces the expression of glucose-6-phosphatase and phosphoenolpyruvate carboxylase-1. Thus, these activities of IGF-1 are not “insulin-like” but rather antagonistic. These findings reinforce the role of IGF-1 in glucose homeostasis. Also, pyruvate dehydrogenase kinase isoenzyme-4 encodes pyruvate dehydrogenase complex (PDK). PDK is an emerging target for the treatment of MetS which may allow the maintenance of the steady-state concentration of adenosine triphosphate during the feed-fast cycle. For that, cells require efficient utilization of fatty acids and glucose, and such is controlled by PDK. Particularly the pyruvate dehydrogenase kinase isoenzyme-4 gene encodes PDK that converts pyruvate, CoA and oxidized nicotinamide adenine dinucleotide (NAD+) into acetyl-CoA, the reduced form of nicotinamide adenine dinucleotide (NADH) and carbon dioxide.

### Insulin-like growth factor-1 and lipid metabolism

IGF-1 promotes preadipocyte differentiation [181], however, as preadipocytes differentiate, they stop expressing IGF-1Rs, delegating such functions now to insulin receptors, which increase in number significantly. Thus, in the adipose tissue, physiological IGF-1 concentrations are not effective in stimulating changes in lipid synthesis or lipolysis, only at high concentrations is capable of stimulating glucose transport via the insulin receptor [182]. Contrarily, a study with 8 GH-deficient subjects found that IGF-1 administration increased lipid oxidation (being this effect more potent when co-administered with GH), energy expenditure, and insulin resistance [108, 183]. This effect is believed to be due to IGF-1 suppression of insulin secretion, which leads to augmented lipolysis in adipose tissue and promotion of FFA use by muscle.

Although mature adipocytes are not a target for IGF-1, they secrete it. In fact, cultured adipocytes secrete more IGF-2 than IGF-1, and predominantly IGFBP-4. Growth hormone, interleukin-β (IL-1β), and TNF-α affect secretion of IGF-1—whereas IGF-2 is affected by TNF-α only. Hence, cytokines may control adipocytes homeostasis by affecting local IGF-1 synthesis [160].

Growth hormone has direct effects on mature adipocytes that result in the release of FFAs following TG breakdown and in increased FFA oxidation in the liver [182]. GH can enhance the lipolytic effect of catecholamines by increasing the number of adrenergic receptors in adipocytes. GH also increases hepatic glucose production [184, 185] and leads to increased lipolysis in adipocytes through the β-3 adrenergic receptor [185, 186]. Such receptor activates the protein kinase A (PKA) cascade, eventually activating lipases [185, 187, 188]. Additional effects include uncoupling of the electron transport chain to produce heat [185, 189]. In skeletal muscle, GH increases lipoprotein lipase activity via the β-3 adrenergic receptor, as a result facilitating FFA use [186].

On the other hand, insulin is a potent stimulant of lipid synthesis, antagonising TG breakdown. An increase in FFA efflux from adipose tissue to liver can result in insulin resistance in the liver and GH is known to antagonise insulin action by this mean [183]. Definitive evidence of the role of FFAs in GH-mediated insulin resistance was obtained in clinical studies in which the effects of exogenously administered GH on insulin resistance were abrogated by Acipimox™—an inhibitor of lipolysis [185, 190, 191]. The increased efflux of FFAs from adipose tissue to lipid-sensitive tissues (such as liver and skeletal and cardiac muscle) increases serine phosphorylation of IRSs. Such phosphorylation leads to the blockade of the tyrosine residues in IRSs, to which the insulin and IGF-1 receptors phosphorylate to activate IRSs and consequently commence the signalling cascade [185, 192], as comprehensively discussed above.

IGF-1 promotes fatty acid transport in muscle [162, 183, 193] and its inhibition causes severe consequences like insulin resistance and eventual diabetes [162]. This is due to the liver taking up all the circulating FFAs, which then interfere with insulin and IGF-1 signalling (as described above) and eventually leading to hepatic steatosis. Therefore, the two major effects that are enhanced by IGF-1 are FFA use by muscle and GH suppression. These two actions result in a decreased FFA flux in the liver, improving insulin and IGF-1 signalling. Such improvement promotes lipogenesis in fat (recall that IGF-1 could maintain cytokine homeostasis—anti-inflammatory effects-, and thus protection from mild inflammation as a consequence of obesity). This fact, linked to the augmented FFA use by muscle and insulin signal reinstitution by IGF-1, results in a marked reduction in total FFA flux.

Moreover, IGF-1 could be implicated in nutrient absorption as our group demonstrated more than a decade ago. We showed that cirrhotic (an IGF-1 deficiency condition) rats had diminished amino acid and glucose intestinal absorption [114, 194, 195] and that IGF-1 replacement therapy was able to restore both alterations, suggesting a role of IGF-1 in the position of transporters [196]. Such findings suggest that IGF-1 implications on metabolism may not only affect energy use and balance, but as well act regulating transport and nutrient absorption. Therefore, this finding could be indicating that IGF-1 deficiency could be altering nutritional balance. However, more profound studies in the matter are necessary.

In the aforementioned study from our group, adult mice with partial IGF-1 also displayed decreased expression of genes involved in lipid metabolism (ATP-citrate lyase, acetyl-CoA acyltransferase 1B, acetyl-CoA acetyltransferase 1) and cholesterol synthesis and transport (Both HMG-CoA reductase and synthase, LDL-related protein 1, proprotein convertase subtilisin/Kesin type 9), resulting in dyslipidaemia. Such genetic alterations were reverted by IGF-1 replacement therapy and may seriously contribute to the establishment of MetS [164].

**Fig. 1:**
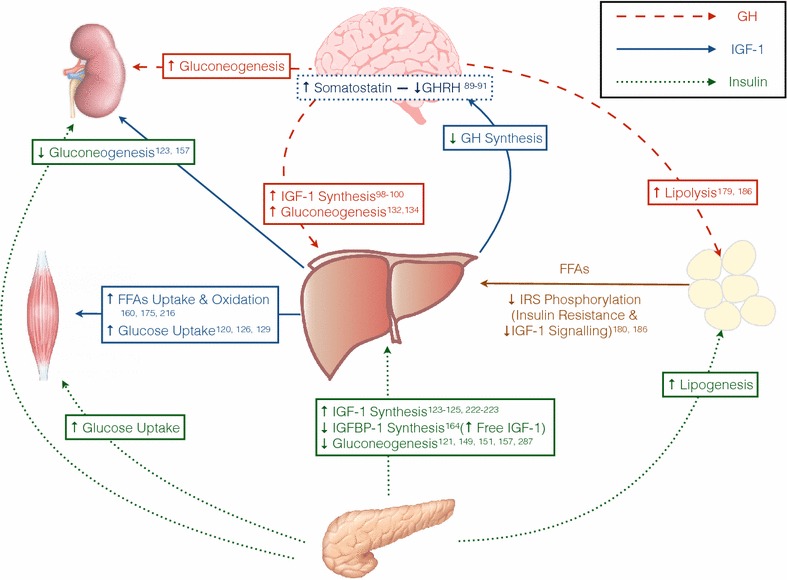
Metabolic effects of IGF-1, GH, and insulin under physiological conditions on their target organs. The figure summarises schematically some of the metabolic effects that IGF-1 (*blue continuous line*), GH (*red discontinuous line*), and insulin (*green dotted line*) exert on kidney (*upper left*), brain (*upper centre*), skeletal muscle (*left*), liver (*centre*), adipose tissue (*right*), and pancreas (*bottom*). *GH* growth hormone, *GHRH* growth hormone releasing hormone, *FFA* free fatty acid, *IRS* insulin receptor substrate, *IGF-1* insulin-like growth factor 1, *IGBBP-1* insulin-like growth factor binding protein 1

Figure 1 and Table 2 have been included to represent schematically IGF-1 actions in metabolism with target organs.

**Table 2:** Metabolic effects of IGF-1

Effect	Mechanism	Experimental model	Reference
IGF-1and lipid metabolism			
Stimulation of preadipocyte differentiation	Through IGF-1R receptor activation	In vitro, in vivo: human	[181, 182]
Stimulation of lipogenesis	IGF-1R stimulation, PPAR-γ involved thought	In vitro	[181, 183]
Lipid uptake and oxidation	Promotion of lipid uptake into the muscle and increased lipid oxidation. Not directly demonstrated. Mechanism not yet elucidated	In vivo: mice	[162, 193]
Insulin secretion suppression	IGF-1 seems to inhibit insulin secretion, thus acting on insulin lipogenic effects on fat	In vivo: human	[138, 140, 197, 198]
Reduction of FFA flux in the liver	By suppressing GH secretion (reduce adipose tissue lipolysis) and by augmented lipid utilisation and oxidation		[162, 193, 198]
Reduction in TG and cholesterol levels	In aging animals. Suggesting that IGF-1 could be involved in aging-related MetS	In vivo: aging Wistar rats	[199]
Decreases fat mass in GH deficient patients	Probably secondary to insulin suppression of insulin-induced lipogenesis	In vivo: human	[197]
Normalise lipid transport	Increasing liver expression of genes: pcsk9, lrp; and reducing gene expression of lpl and fabp5	In vivo: Hz (igf^+/−^) mice with partial IGF-1 deficiency	[164]
Restore lipid metabolism	Increasing liver gene expression of acaa1b, acat1, hmgcst1, hmgrc; reduced in mice with partial IGF-1 deficiency and reverted by replacement therapy	In vivo: Hz (igf^+/−^) mice with partial IGF-1 deficiency	[164]
IGF-1 and carbohydrate metabolism			
Augments energy expenditure	By improving mitochondrial function and protection, thus being able to produce ATP more efficiently with an O/P ratio improved, oxidative damage reduction, protein damage reduction, and calcium handling improvement	In vivo: mice, rats and humans	[105, 106, 108, 200]
Glucose uptake	In muscles through actions on IGF-1R and hybrid receptors	In vitro, in vivo: mice, rat	[133, 161, 162, 171, 172, 201]
	In all peripheral cells through IGF-1R, insulin, and hybrid receptors	In vivo: mice, rat, human	[137, 147, 202– 205]
	Increases placental basal membrane content of GLUT-1	In vitro	[206]
Suppress renal and hepatic gluconeogenesis	High [IGF-1] through its IGF-1 own receptor and hybrid receptors	In vivo: mice, human	[163]
Enhancement of insulin sensitivity and actions	Not only through GH suppression, but IGF-1 directly aiming IR actions through IGF-1R and hybrid receptors	In vitro, in vivo: mice, human	[107, 153, 155, 161, 162, 165– 167, 203, 207– 209, 210, 211]
Increases sugar intestinal transport	Probably by direct effect on enterocyte cytoskeleton, restoring normal position of transporters	In vivo: cirrhotic rats In vitro: in BBV from cirrhotic rats	[110, 194, 195]
Enhances carbohydrate oxidation in patients with GH receptor mutations	Physiologic replacement of IGF-1 improved carbohydrate oxidation	In vivo: humans	[166]
Increases hepatic glucose production in patients with GH receptor mutations	By suppression of insulin, but maintaining overall normoglycaemia	In vivo: humans	[166]
Glucose homeostasis gene modulation	Restores liver gene expression of g6pc, pck1, pdk4, and acly; all them reduced in heterozygous mice with partial IGF-1 deficiency	In vivo: Hz (igf^+/−^) mice with partial IGF-1 deficiency	[164]

*IGF-1* insulin like growth factor 1, *PI3K* phosphatidylinositol-4,5-bisphosphate 3-kinase, *AKT* protein kinase B, *GLUT1*glucose transporter 1, *PC* pyruvate carboxylase, *PEPCK* phosphoenolpyruvate carboxykinase, *FFA* free fatty acids, *acaa1b* acetyl-CoA acyltransferase 1B, *acat 1* acetyl-CoA-sinthetase 1, *acly* ATP-citrate lyase, *fabp1* fatty acid binding protein 1, *fabp 5* fatty acid binding protein 5, *g6pc* glucose-6-phosphatase, *pck1* phosphoenolpyruvate-carboxilase, *hmgcst* 3-hydroxy-3-metilglutarilCoA-sinthetase, *hmgrc* 3-hydroxy-3-methylglutaryl-CoA reductase, *lpl* lipoprotein lipase, *lrp* low density lipoprotein receptor-related protein 1, *pcsk9* proproteinconvertase subtilisin/kexin type 9, *pdk4* pyruvate deshydrogenase kinase isoenzyme 4

## Can insulin-like growth factor-1 deficiency be involved in metabolic syndrome establishment?

An assortment of epidemiological and clinical studies have stated glucose and lipid metabolism alterations, insulin resistance, and central obesity as predominant factors for the development of MetS [38, 212].

**Fig. 2:**
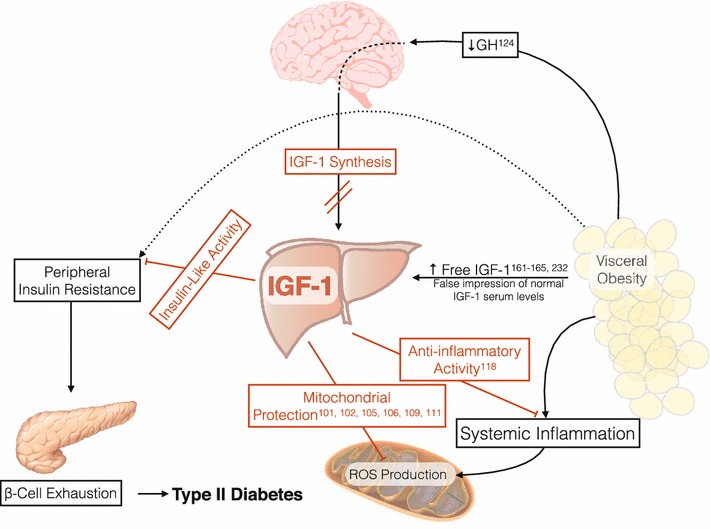
Metabolic effects of IGF-1 and GH under pathological conditions. The figure summarises schematically some of the metabolic mechanisms altered in obesity and the role that IGF-1 and GH exert on them

Similarities between insulin and IGF-1 suggest the possible role of IGF-1 in the pathological process of this syndrome, therefore several studies have attempted to correlate IGF-1 plasma levels with MetS. Figure 2 represents the pathophysiology of an altered IGF-1/GH/insulin axis, and potential beneficial actions of IGF-1 therapy.

A general finding is that obese patients fulfilling criteria for MetS together with low IGF-1 plasma levels tend to develop a worse cardiovascular disease outcome than those with mid-normal to high-normal IGF-1 levels [213]. Nevertheless, many of them also present insulin resistance and inflammatory cytokine secretion, so it is difficult to determine the exact role of each component in the cardiovascular outcome.

Nonetheless, low IGF-1 circulating levels are also associated with reduced insulin sensitivity [207], glucose intolerance, and T2D [207, 208, 214]. Moreover, some inflammatory cytokines are known to reduce IGF-1 levels in animal models [215]. Additionally, the IGF-1/IGFBP-3 ratio, a common rough of free IGF-1 levels, is significantly decreased in obesity [216], however no IGF-1 bioactivity was estimated. This parameter has been further studied, showing that those men and women in the lowest quartile of the IGF-1/IGFBP3 ratio are threefold more likely to meet the Adult Treatment Panel III (ATP-III) definition for MetS, and twice as likely to be insulin resistant—that IGF-1/IGFBP-3 ratio decreases notably as the number of MetS components increases [217]. Furthermore, visceral adipose tissue mass has been inversely correlated with circulating IGF-1 levels [216]. Nevertheless, the mechanism of this possible inverse relationship between MetS and free IGF-1 levels remains unclear.

A very interesting epidemiological study that supports this idea showed that in normal subjects, IGF-1 contributes to glucose homeostasis. This study analysed a group of Dutch Caucasians with a polymorphism in the promoter of the IGF-1 gene [129, 218]. Results within this group showed a reduced IGF-1 secretion—40 % lower than those without the polymorphism. These subjects are 2.1 cm shorter and have 2.2-fold increase in T2D prevalence after the age of 60 [129, 219]. A different study [220] tested IGF-1 in response to energy intake in the Gujarati migrant community in Sandwell (UK) and data was compared with people still resident in their village of origin in India. Total energy and total fat intake were higher in UK migrants, as were IGFBP-3 and IGF-1, but IGFBP-1 was lower in UK migrants. At both sites, IGF-1 and IGFBP-3 correlated positively with total energy and fat. Conversely, in Indian Gujaratis, IGFBP-1 fell with increasing total energy and fat intake but not in UK Gujaratis.

Several other studies (one of them being a large scale community-based Framingham Heart Study [221]) have suggested a role for IGF-1 in the prevalence of insulin resistance and MetS [221–223]. Biomarkers correlating the lower IGF-1 concentration to increased waist-to-hip ratio or to impaired glucose tolerance are also being studied [224, 225].

Insulin-like growth factor-1, as discussed earlier, has implications on lipid and glucose metabolism [87, 88], and its exogenous administration enhances insulin sensitivity in healthy adults [139, 140] as well as those with T2D [155].

A fascinating study including over 500 patients revealed that IGF-1 concentrations were independently associated with insulin sensitivity accounting for 10.8 % of its variation. The results were assessed by HOMA-S together with anthropometric measurements, HDL, TG and blood pressure, and found correlations between these parameters and IGF-1 plasma levels. Additionally, they established that according to the WHO definition for MetS, each unit increase in log-transformed IGF-1 concentrations, was associated with a 90.5 % reduction in the risk of MetS [226].

Salmon et al. [227] showed that transgenic mice with reduced levels of IGF-1 can induce female insulin resistance [227]. Moreover, global deletion of IGF-1 gene expression in mice does not result in glucose intolerance. It has to be mentioned that KO mice for IGF-1 gene are not viable and studies in such mice have to be done in the first days of life, and thus results are not very conclusive as metabolism is not properly established at this stage [129, 228]. However, if a partial deletion is present, the mice will develop glucose intolerance if starved. Additional studies found that elimination of hepatic IGF-1 gene expression results in a compensatory threefold increase in GH secretion—recall that IGF-1 is secreted by GH stimulation in hepatocytes. This combination of lowered serum IGF-1 and increased GH secretion leads to increased insulin resistance—as in the systemic deletion but also developed glucose intolerance [129, 209]. Interestingly, glucose intolerance could be improved when IGF-1 was systemically administered. This response was caused primarily by GH hypersecretion, as expression of a GH antagonist resulted in improvement of glucose homeostasis [129, 229]. Additionally, administration of IGF-1 in the presence of this antagonist results in a further improvement in insulin sensitivity; suggesting that, at high concentrations, IGF-1 has effects not simply mediated by suppressing the effect of GH on hepatic insulin sensitivity [129, 209]. In a similar study, the pivotal role for the IGF-1 in insulin sensitivity has received further support from liver-specific IGF-1 KO mice which exhibited overt insulin resistance and hyperinsulinaemia that was reversed by the administration of IGF-1 [161].

Moreover, results from the aforementioned Framingham heart study also demonstrated the correlation between low IGF-1 and the increasing metabolic syndrome markers [221]. A good example is the finding that low circulating levels of IGF-1 are independently associated with hyperglycaemia and insulin resistance in adults [160, 230–232]. To the contrary, high to normal levels of circulating IGF-1 correlate with a rise in adiponectin levels and a reduced prevalence of MetS is found [233].

Since the liver is the major site of IGF-1 production, when steatosis develops lowering insulin sensitivity, the severity of steatosis at different stages of insulin resistance and metabolic syndrome seems to be correlated with worsened circulating IGF-1 levels [234]. In addition, low IGF-1 subjects in a study were found to possess up-regulated fatty acid metabolism along with down-regulated GLUT-1 gene (in charge of glucose uptake in erythrocytes, brain endothelial cells, eye, peripheral nerve and also responsible for materno-placental glucose transfer) [210].

In summary, reconciling all discussed aspects relevant to insulin, IGF-1 improves insulin sensitivity by suppressing insulin and GH secretion and by improving insulin signalling indirectly reducing FFA flux.

When we look to IGF-1 levels in sera from T2D patients, the results found are very wide [235]. It must be considered that multiple factors interact to control IGF-1 levels, many of which are disturbed in T2D, namely: increased inflammatory cytokines, decreased hepatic insulin action due to resistance, concomitant changes in IGFBPs, and the effects of obesity. In addition, T2D is the result of a complex interaction of environmental and genetic factors, being difficult to establish the role of each one in the pathogenesis of diabetes and in the levels of IGF-1. In an experimental model, transgenic mice expressing a kinase-deficient IGF-1R β-subunit (thus displaying reduced signal transduction in both IGF-1R and hybrid receptors) developed diabetes early on life [162, 236]. Also, mice carrying a genetic mutation that lack one of the *igf1r* alleles (*igf1r*^+/−^) show a 10 % reduction in post-natal growth, insulin resistance and glucose intolerance [237]. Additionally, infants born small for gestational age who exhibit low IGF-1 levels presented a higher risk for the onset and development of T2D in adult life than those born with normal weight [238, 239]. Nevertheless, it is noteworthy that the inverse correlation between IGF-1 and diabetes only prevails in younger individuals (<65 years) [231], establishing that this deficiency can lead to MetS—as aging can be considered an IGF-1 deficiency condition [107].

Abnormal IGF-1 and GH levels have been proposed to play a key role in obesity [130, 240, 241]. Obese human and animal models are generally accompanied with abnormal circulating IGF-1 levels [130], as well as IGF-1/IGFBP-3 ratio; and an inverse relationship between IGF-1 and visceral fat mass distribution has been described [242–246] as previously mentioned.

Moreover, some studies revealed that high fat diet promoted reactive oxygen species (ROS) and cytokine production, apoptosis, protein and mitochondrial damage, and reduced ATP content. These defects were accompanied by disrupted phosphorylation of the IRS, as well as down-regulated expression of mitochondrial proteins PPARγ co-activator 1α (PGC1α) and uncoupling protein-2 (UCP-2) [200]. All of these factors can be alleviated by the cytoprotective and anti-inflammatory actions of IGF-1 [105, 123, 247–249].

The culmination of all of these studies come to light with the finding of several (some reported and many from our group still publication pending) Laron’s Syndrome (congenital IGF-1 deficiency or GH insensitivity) never treated patients who underwent progression to MetS and ultimately to T2D together with diabetic retinopathy when they reached their late 30 s, [250, 251].

### The role of insulin-like growth factor binding proteins in metabolic syndrome

Recall that IGFBPs have a very important role, not only in modulating free IGF-1, half-life and localisation, but also by possessing IGF-independent activities mediated by their own receptors. Changes in IGFBPs have been correlated with certain parameters of MetS. For instance, low IGFBP-1 levels with high C-reactive protein values are strong predictors of MetS [252, 253]. Low IGFBP-2 also acts as a good marker for MetS along with high fasting glucose [254]. Even though the data is extensive it is not enough to explain any relationship between these molecules and the independent MetS factors, or any mechanism by which these molecules could be involved in the pathophysiology.

As a result of IGF-1 changes, IGFBPs are also altered in T2D [255]. Studies in prediabetes suggest that IGFBP-1 shows normal levels before developing T2D [256]. This may be caused by hyperinsulinaemia during the onset of the disease. Elevated insulin causes an increase of free serum IGF-1, nevertheless, as resistance to insulin progresses, the liver becomes insensitive to insulin-mediated suppression of IGFBP-1 [254, 256]. On the other hand, IGFBP-3 undergoes augmented proteolysis on diabetic patients which results in a sudden rise of free IGF-1 concentrations [257, 258].

Overexpressed IGFBP-1 in transgenic mice shows that its increase produces hyperinsulinaemia accompanied by glucose intolerance, and that this process is dependent upon IGFBP-1 phosphorylation state—which is increased in diabetes [259]. In another study it was found that after weight loss there is a rise in IGFBP-1 and IGFBP-2, both in children and adults [260, 261]. Considering that IGF-1 regulates IGFBP-1 and IGFBP-2, it is reasonable to conclude that early changes in insulin resistance alter their concentrations.

A recent study tested whether IGFBP-1 concentrations were able to predict the development of T2D in women. The outcomes showed that women with the lowest fasting IGFBP-1 at baseline had a higher risk for developing diabetes within 8 years, also showing an impaired IGFBP-1 suppression after oral glucose loading [256]. Conversely, in another study, 615 patients with IGF-1 values in the lower half of the normal range were closely observed during 4.5 years. It was found that there was an increased predisposition to develop glucose intolerance or T2D, and that this change was independent from IGFBP-1 [208].

Moreover, IGFBP-2 is decreased in obese patients and thus increasing free IGF-1 [262]. This fact is relevant as transgenic mice overexpressing IGFBP-2 were resistant to developing obesity when fed with high fat diet [174], suggesting that this molecule could have a direct effect on preadipocyte differentiation.

### IGF-1 treatment: future and limitations

The FDA approved the use of rhIGF-1 (Mecasermin, Increlex™) for treatment of severe primary IGF-1 deficiency in 2003. By the same time, the FDA approved the use of a equimolar combination of IGF-1 and IGFBP-3 (Mecasermin Rinfabate, iPlex™), suggesting that it would be a better choice, seeming as it will require lower doses because of the augmented half-life of the molecule and “buffering” effect on concentration. Additionally this complex can bypass the IGF-2 displacement effect. Because IGFBP-3 also carries IGF-1, when IGF-1 is administered on its own, the carrier IGFBP-3 saturates with IGF-1 displacing IGF-2 and thus augments its free circulating concentration. However, subsequent studies revealed that no significant difference (among with some patient issues) could be observed, so now IGF-1 alone seems to be an efficient treatment. Recombinant human IGF-1 is usually synthesised in *E. coli* and subsequently purified. Its purification is a very insidious process that elevates the cost, and this fact in combination to the limited applicability that has been linked to date, elevates the price of the treatment.

The use of analogues has shown multiple and substantial clinical benefits along with metabolic improvement. A promising therapeutic approach being studied uses IGF-1 analogues such as PEG-IGF-1 [263], which offers promising protection against acute contraction-induced muscle injury.

To date several clinical trials have been conducted to test IGF-1 under several conditions and an infinity of animal models have been used to investigate its deficiency, treatment and to exploit its actions. Phase I studies from the late 1980s using IGF-1 to treat Laron’s Syndrome (GH insensitivity) revealed effective increase in linear growth reaching adult heights using doses ranging from 80 to 240 µg/kg/day [264–268]. Different groups of patients and candidates for IGF-1 treatment have been those with an IGF-1 gene deletion and others with idiopathic short stature. Furthermore, phase II studies were undertaken to prove the efficacy of mecasermin rinfabate (IGF-1/IGFBP-3) in paediatric severe burns and had promising outcomes when doses of 1–4 mg/kg/day [269–273] were used. Moreover IGF-1 was tested in patients with osteopenia/osteoporosis linked to anorexia nervosa and severe bone fractures with positive results utilising doses ranging from 30 µg to 1 mg/kg/day for 2–9 months [274, 275].

When treating metabolic disorders with IGF-1, phase II trials until now conducted by Clemmons et al. including adult patients with either T1D or T2D treating with mecasermin rinfabate (1–2 mg/kg/day for 14 days) showed lowered exogenous insulin requirements while improving glycaemic control. These results suggest improvement in insulin sensitivity, and moreover, no additional side effects to those found with placebo were observed in T1D, meanwhile in T2D patients oedema, jaw pain and arthralgias were 4 % lower than previous reports [276, 277]. Evidence revealed that endogenous and exogenous IGF-1 administration protects against the onset and progression of diabetic cardiomyopathy [278, 279]. Consistent with this, patients with T2D responded to IGF-1 treatment with improved glucose tolerance, hyperinsulinaemia, and hyperlipidaemia as previously stated [280]. Up to date, limited information is available regarding obesity treatment by regulation of the GH-IGF-1 system. Apart from all of these conditions tested under IGF-1 treatment, there are a number of other ones, among others: cystic fibrosis, AIDS, Chron’s, multiple sclerosis and ALS—which are carefully revised by Rosenbloom [281].

Despite all the above-mentioned clinical benefits, the safety of long-term administration of this hormone remains controversial. There is supporting evidence reporting adverse effects from long-term rhIGF-1 treatment including neoplastic formation, cataract and renal hypertrophy, all of which seem to be the most severe effects observed and are usually rare [123, 282–284]. Additionally these complications were transient, easily treated and tolerated without treatment discontinuation. More common side effects within the mentioned studies revealed mild to moderate effects like pain at site of injection or headache that was transient and disappeared after 1 month [268]. Other side effects reported ranged from lipohypertrophy at injection site, papilloedema (related to cranial hypertension), and facial nerve paralysis [276, 285], however symptoms did not prevail after treatment pause and restarting with lower doses [268]. The usual concern with regards to treatment with IGF-1 has historically been hypoglycaemia, however in the trials done so far it has not always occurred and had been lessened when administered with meals—it was also usually connected to an appearance in a loss of appetite [264, 286]. Another reported effect has been growth of lymphoid tissue (specially acromegaly and tonsillar hypertrophy), renal enlargement (with normal kidney function) and in rare cases facial coarsening of features and incremented hair growth [264].

Of further importance is the well-known effect of exercise in IGF-1 plasma level rise. It has been determined for years that after only single bouts of moderate to high-intensity total IGF-1 plasma levels rise up to ∼10–30 % and peak only after 5–10 min after the onset of the training [287]. This rapid increase has been argued to be due either to a release of IGF-1 stores in tissues or due to a proteolytic cleavage of IGFBP-3 either in the plasma or in tissue. Also it must be noted that during exercise pH drops dramatically and this affects negatively to IGFBPs affinity for IGF-1/2. It has been somehow elucidated that muscle tissue is the one movilising most of the IGF-1 from cells to plasma [288]. This muscle autocrine/paracrine/endocrine effect has various effects. Apart from the obvious differentiation of satellite cells, which may account for many of the beneficial actions of exercise training, without excluding the classical fat burning, cardiovascular tonification, neuroendocrine system activation and chronic inflammation grade lowering. These actions have been reviewed herein, but of special mention during exercise is the recruitment of hippocampal neuroprogenitor cells and improved neuroglia function [289, 290]. Moreover, it has been recently proven that IGF-1 central administration also induced improvement in insulin sensitivity [291], meaning that this sudden rise after exercise or treatment could be of benefit not only in the periphery, but acting centrally. It must be reminded that in our experience, IGF-1 treatment does not only raise IGF-1 plasma levels, but also stimulates tissues to synthesise their own IGF-1 by a mechanism still not uncovered, aiming these mechanisms of central stimulation of peripheral improvement. These facts are in accordance with the perspective of this review which suggests the central and nuclear role of IGF-1 in metabolism. Because exercise is the current best option when it comes to restore metabolism and obesity inflammation problems (i.e. for MetS and T2D), and because exercise is one of the most potent IGF-1 synthesis/freeing mechanisms, it seems logical to correlate them and establish IGF-1 as a target for future options in the multifactorial treatment for metabolic syndrome.

Inasmuch, these complications have caused fear and controversy among studies and opinions towards the safety of the treatment even when there are findings supporting the need for further investigation in the field of metabolic disorders. In our experience, and the problem we often see in these trials, comes to the dosage administered which are brutally over the target of restoring the physiological normal levels of the hormone. All studies have been conducted using 80 µg–4 mg/kg/day and ranging in a wide spectrum of lengths (weeks to months) and routes of administration. In all our previous murine studies we have used short cycles (10–14 days) of very low subcutaneal doses (20 µg/kg/day) after carefully assessing its circulating concentrations and using the minimum amount that was sufficient to adequately restore physiological values of the molecule. Using these concentration we have not yet perceived any of the reported adverse affects, including hypoglycaemias, retinopathies or any others. We are conscious of the limitations of the animal models [123], however in a clinical trial undertaken by this group to asses IGF-1 in liver function under cirrhosis with these same dosage (20 µg/kg/day) no side effects were reported and liver function greatly increased [108]. So, as this group has been experimenting over the last years, the problem with IGF-1 is a matter of dosage. Even more intriguing is the fact that none of the inclusion criteria for the above-mentioned studies encompassed tumour markers to potentially discard any ongoing or potential tumour process that, obviously, IGF-1 could accelerate. IGF-1, as happens with other hormone therapies (thyroid hormone or insulin), should never been used lightly without firstly assessing its deficiency (local, central or systemic) and secondly by ascertaining that no tumours are near to be generated. Careful monitoring during treatment should be carried out to guarantee a safe outcome.

## Conclusions

It can be plainly concluded that everyday more IGF-1 roles are being unravelled which concern energy metabolism. In the present review we have summarised how the GH/IGF-1 axis, together with insulin and IGFBPs act in a coordinated manner to regulate energy flux and use. From our point of view, when this whole system becomes altered-whether obesity, genetics or environmental factors act to disrupt its symphony-insulin resistance, steatosis, MetS, and ultimately T2D may develop. In our experience and from the information presented in this review, IGF-1 acts as the cornerstone maintaining homeostasis in this system.

In the last decade several studies have been conducted which revealed the relevant role of IGF-1 in the development of MetS. Briefly, an inverse correlation between IGF-1 (IGF-1/IGFBP-3 ratio) circulating levels and several markers for obesity, MetS, T2D, and CVD has been found; and thus, it could indicate that low circulating levels of IGF-1 can lead to MetS and raise the risk for CVD and T2D. Nevertheless, more studies are needed to describe the exact mechanism by which IGF-1 impacts and interacts with other factors and hormones in order to develop each component of MetS, T2D and its cardiovascular consequences.

The axis GH/IGF-I is claiming a particular physiological understanding. Usually IGF-1 deficiency is associated to “GH resistance” or “GH insensibility” states. IGF-1 replacement therapy induced a restoration of the altered GH/IGF-1 axis by reducing circulating GH levels and recovering the somatostatinergic tone [292].

Low doses of IGF-1 seem to be able to restore circulating levels of this hormone promoting beneficial effects without undesired side effects (including hypoglycaemia). Secondary effects from IGF-1 therapy were reported after administration of doses higher than 60–80 μg/kg/day.

In summary, the implication of IGF-1 in glucose and lipid metabolism and its derangements when the GH/insulin/IGF-1 axis balance fails is well documented. IGF/IGFBPs level alterations in MetS have been, to some extent, observed, however the exact mechanism has not been elucidated—still it suggests a trend towards IGF-1 local or systemic deficiency or availability. It is still too early to consider IGF-1 as the key factor; rather, literature is providing the clue suggesting that it is a good endeavour to pursue in order to achieve an appropriate pharmacological target that could aim at reversing MetS in parallel with diet and exercise before it onsets T2D. This group only suggests such treatment when IGF-1 deficiency prevails, acting merely as a substitutive therapy, and never to exploit its multiple actions. Diet and exercise must be considered as the first line of “treatment”, however if they fail to correct metabolic signalling, IGF-1 therapy seems to be promising at metabolic harmonisation. For this to become a fact, however, further carbon tracer, protein and gene expression studies, together with large patient protocols are needed to reveal the exact role of IGF-1 in the whole system; meaning, to establish a way to direct IGF-1 supplementation towards exactly where it is needed in order to avoid MetS from progressing to more grave outcomes.

## Abbreviations

MetS: Metabolic syndrome
IGF-1: Insulin-like growth factor
CVD: Cardiovascular disease
T2D: Type 2 diabetes
RAS: Protooncogene protein P21
NAFLD: Non-alcoholic fatty liver disease
OSA: Obstructive sleep apnea
HDL: High density lipoprotein
TG: Triglyceride
PI3K: Phosphatidylinositol-4,5-bisphosphate 3-kinase
AKT: Protein kinase B
GLUT: Glucose transporter
FA: Fatty acid
FFA: Free fatty ACID
IGF-1R: IGF-1 receptor
IGFBP: Insulin-like growth factor binding protein
IRS: Insulin receptor substrate
KO: Knock out
LDL: Low density lipoprotein
VLDL: Very low density lipoprotein
GH: Growth hormone
HOMA: Homeostasis model assessment
ROS: Reactive oxygen species
